# Measurement of diabetes-related emotional distress using the Problem Areas in Diabetes scale: psychometric evaluations show that the short form is better than the full form

**DOI:** 10.1186/s12955-014-0142-z

**Published:** 2014-10-29

**Authors:** Eun-Hyun Lee, Young Whee Lee, Kwan-Woo Lee, Yong Seong Kim, Moon-Suk Nam

**Affiliations:** Graduate School of Public Health, Ajou University, 164 Worldcup-ro, Yeongtong-gu, Suwon-si, 443-380 Gyeonggi-do Republic of Korea; Department of Nursing, Inha University, Incheon, Republic of Korea; Department of Endocrinology and Metabolism, School of Medicine, Ajou University, Suwon, Republic of Korea; Division of Endocrinology & Metabolism, School of Medicine, Inha University, Incheon, Republic of Korea

**Keywords:** Diabetes, Emotion, Distress, Psychometric properties, Translation

## Abstract

**Background:**

The Problem Areas in Diabetes (PAID) scale is widely used for measuring diabetes-related emotional distress. There has been debate over the last 2 decades about the underlying factorial-construct validity of the PAID, with one- to four-factor structures being reported. A short form of the PAID, which comprises five items (PAID-5), was recently developed using Western patients with type 2 diabetes. This study measured the psychometric properties of the full and short forms of the PAID in Korean patients with type 2 diabetes, with the aim of determining which form is preferable.

**Methods:**

The PAID and PAID-5 were translated into Korean (K-PAID and K-PAID-5, respectively) using a forward-and-backward translation technique. The study participants were recruited from university hospitals. The factorial-construct, convergent, and known-groups validity, and internal-consistency and test–retest reliability of both the K-PAID and K-PAID-5 were evaluated.

**Results:**

For the K-PAID, confirmatory factor analysis revealed a marginal fit to the one-, two-, three-, and four-factor models. The three- and four-factor models of the K-PAID partially satisfied the internal-consistency and test–retest reliability, and convergent and known-groups validity. For the K-PAID-5, confirmatory factor analysis demonstrated an excellent fit to the one-factor model, with a Cronbach’s alpha of 0.87 and an intraclass correlation coefficient of 0.89. The K-PAID-5 satisfied the convergent validity, as evaluated using the Center for Epidemiologic Studies Depression Scale and hemoglobin A1c. Known-groups validity by gender was also satisfied.

**Conclusions:**

The K-PAID-5 demonstrated excellent psychometric properties as a one-factor scale. The brevity of the K-PAID-5 represents a major advantage in a practical context in that it may impose a minimum burden upon patients with diabetes.

## Background

Diabetes is a global health problem that affects about 382 million people worldwide, with the number being expected to rise to 592 million by 2035 [1]. Patients with diabetes require complex and lifelong self-management, including diet, physical exercise, medication, and blood-glucose monitoring. Many patients experience emotional burdens in response to these prolonged requirements, including worry about complications, fear of hypoglycemia, feeling of guilt regarding uncontrolled blood glucose, and depressed mood [2]. These negative emotional burdens associated with diabetes are defined as diabetes-related emotional distress [3]. It has been reported that this emotional distress is a negative predictor of medical outcomes such as blood-glucose control and health-related quality of life [3-5]. Therefore, the American Diabetes Association and the International Diabetes Federation have recently recommended that clinicians routinely and effectively assess their patients’ level of emotional distress using appropriate tools such as validated questionnaires [6,7].

The Problem Areas in Diabetes (PAID) scale is a 20-item representative self-reported instrument for measuring diabetes-related emotional distress, and covers a range of negative emotional problems of patients with diabetes [3]. This instrument was originally developed in the USA for patients with diabetes. The United States Food and Drug Administration noted that the psychometric properties of patient-reported outcome (PRO) instruments such as the PAID need to be satisfied [7]. Although the PAID is a widely used PRO instrument in research, the last 2 decades has been characterized by unresolved confusion and debate regarding its underlying factorial-construct validity. The dimensionality of the PAID was not explored in the original psychometric study [3], although some of the original developers subsequently demonstrated that it had a one-factor structure [8]. However, there has been as yet no consensus regarding the factor structure of this tool, with one- to four-factor structures being reported for diabetic populations in the Netherlands/USA [9], Sweden [10], Iran [11], Greece [12], USA [13], Iceland [14], Turkey [15], and Taiwan [16].

The lack of consensus regarding the factor structure of the PAID may simply be attributed to the differences across cultures or countries with respect to the experience of emotional distress. However, that alone is not sufficient to account for the discordance, since some of the psychometric studies performed in different cultures yielded the same one-factor structure [8,16,17], while others conducted within the same country yielded different underlying PAID structures [8,13,16,18]. Indeed, none of the factor structures was supported empirically in a study conducted in Norway [19]. These findings imply that some other parameter beyond the patients’ cultural background may have determined the different factor structures of the PAID between studies.

One potential determinant could be the statistical method used to analyze the study findings. Huang et al. [16] suggested that the inconsistent results regarding the PAID factor structure were due to the use of exploratory factor analysis (EFA), as employed in most of the previous studies. In fact, EFA is useful for reducing the number of items of a scale with a minimum number of dimensions. If a prior hypothesis exists based on a theory or empirical evidence, confirmatory factor analysis (CFA) is more appropriate for identifying a scale’s underlying structure [20]. Another possible determinant could be the medical characteristics of the populations studied. Sigurdardottir and Benediktsson [14] emphasized that the PAID factor structure extracted from a study of patients with type 1 diabetes might differ from that extracted from studies involving only patients with type 2 diabetes or patients with either type 1 or 2 diabetes. Therefore, the PAID factor structure needs to be clarified using CFA in a population that is controlled for the type of diabetes.

A 5-item short form of the PAID (PAID-5) was recently developed using Western patients (mainly Europeans) with type 2 diabetes, with the items selected from the original pool of 20 items [21]. This short form of the instrument requires further psychometric testing, especially in Asian populations. If the psychometric properties of the PAID-5 are equivalent to those of the full-form PAID, it would be preferable to apply it in both practice and research. However, to the best of our knowledge a comparison of the psychometric properties of both the full and short forms of the PAID has yet to be conducted using the same sample.

After reflecting upon the aforementioned problems, this study evaluated the psychometric properties of both the full- and short-form PAID using as subjects Korean patients with type 2 diabetes, with the aim of determining which form is preferable.

## Methods

### Design and sample

The participants for this study were recruited using a convenience sampling method from outpatient clinics at two university hospitals in the Republic of Korea. The following eligibility criteria for participation were applied: a diagnosis of type 2 diabetes, age at least 20 years, and articulate in the Korean language. This study was approved by the institutional review boards at the participating university hospitals prior to commencing data collection. Research assistants informed the patients regarding the purpose of the study, that they could withdraw at any time, and about data confidentiality, after which the patients provided written consent to participate. The participants were then asked to complete a package of questionnaires in a room at the outpatient clinics.

A total of 440 patients aged between 20 and 82 years (58.02 ± 10.88 years, mean ± SD) participated in this study. Approximately half of them were male (*n* = 226, 51.4%) and employed (*n* = 216, 49.1%), and most of them were married or living with their partner (*n* = 351, 79.8%). The duration of diabetes among the entire cohort was 10.66 ± 7.29 years, and the numbers of patients taking an oral hypoglycemic agent (OHA), taking an OHA in combination with insulin, taking insulin only, and managing their diabetes by diet/exercise alone were 292 (66.4%), 112 (25.5%), 20 (4.5%), and 16 (3.6%), respectively.

A subgroup of the participants (*n* = 109) who had completed the package of questionnaires in the outpatient clinics were invited to complete the questionnaires again to assess their test–retest reliability. Of these, 70 (64.2%) patients agreed to participate in the repeated measurements. A stamped, self-addressed return envelope was enclosed with the questionnaires and given to these participants, who were asked to take them home, complete them 10 days later, and then return them by post in the envelope provided via a post office near their home. Research assistants sent them cellular-phone text-message reminders to complete and return the questionnaires. A final total of 58 (82.8%) patients returned the packages via regular mail.

### Instruments

#### Translation of the PAID and PAID-5 into Korean

The original version of the PAID was developed in English and comprises 20 items [3]. Each item was originally scored on a 6-point scale, but this was later modified to a 5-point Likert scale ranging from “not a problem” (score of 0) to “serious problem” (score of 4) [22]. The sum of the 20 items is multiplied by 1.25 to produce a final possible score of 0–100, with higher scores indicating greater diabetes-related emotional distress. With only 5 items selected from the original pool of 20, the PAID-5 comprises a single dimension (i.e., one factor) with items 3, 6, 12, 16, and 19. The possible total scores of the PAID-5 range from 0 to 20, again with higher scores implying greater emotional distress [21].

The English version of the PAID was translated into Korean using a forward-and-backward translation technique that focused on semantic equivalence [23]. Two bilinguals participated in the forward translation, and two other bilinguals independently performed the backward translation. The final translated version was produced in consensus meetings involving three experts in clinical practice or psychometrics.

Interviews were then conducted to explore the comprehensibility of the translated items in 20 patients with type 2 diabetes with the following characteristics: age 59.50 ± 11.67 years, 12 (60%) males and 8 (40%) females, duration of diabetes of 7.29 ± 5.24 years, and 13 (65%) with an educational level of high school or above. Six patients (30%) found that item 1 (“Do not have clear and concrete goals for my diabetes care”) and item 2 (“Feel discouraged with my diabetes treatment plan”) were difficult to understand, and so the panel revised these items after discussion. A Korean language and literature professor then checked all the items on the scale, and reported that item 19 in the Korean version (“Coping with complications of diabetes”) did not project a negative sense, whereas the other items did contain negative meanings. Therefore, the item was revised by the panel and the linguist. As with the English version of the PAID-5, the Korean version (K-PAID-5) comprises five items (items 3, 6, 12, 16, and 19) from the pool of 20 items in the translated Korean version of the full-form PAID (K-PAID).

#### Center for epidemiologic studies–depression scale

The convergent validity of the K-PAID and K-PAID-5 was tested using the hypotheses that their scores would be moderately correlated with depressive symptoms [19]. The Center for Epidemiologic Studies–Depression scale (CES-D) was used to measure the depressive symptoms [24]. Twenty items of that scale are rated on a 4-point Likert scale, with total scores ranging from 0 to 60, and higher scores indicating greater depressive symptoms. The psychometric properties of the CES-D have been validated in a Korean population [25].

#### Hemoglobin A1c

Glycated hemoglobin A1c (HbA1c) levels were used to assess the convergent validity of the K-PAID and K-PAID-5, since greater diabetes-related emotional distress is reported to be significantly correlated with poor glycemic control (*r* = 0.15–0.34) [12,16,26]. The HbA1c levels, which were determined within 3 months prior to the beginning of this study using high-performance liquid chromatography at the two university hospitals involved in this study, were collected from the patients’ medical records.

#### General characteristics of the patients

The patients’ demographic characteristics (age, gender, marital status, and employment status) were obtained using a self-reported survey. Medical characteristics (duration of diabetes and treatment regimen) were collected from their medical records. The general characteristic of gender was used to test the known-groups validity of both the K-PAID and K-PAID-5, since the scores are known to be higher for women than for men [9,15,21].

### Statistical analysis

The SPSS Statistics (version 18) software was used to analyze the data. The quality of the data was assessed using the percentage of item-missing values and item-mean scores of the K-PAID and/or K-PAID-5 with descriptive statistics. It was considered that an item with ≥15% of missing values was not acceptable [27]. Factorial-construct validity was tested using CFA with a maximum-likelihood estimation method. The model was considered to adequately fit the data when the following criteria were satisfied: ratio of the *χ*^2^ value to the degrees of freedom (CMIN/DF) <3.0, goodness-of-fit index (GFI) >0.90, comparative-fit index (CFI) >0.90, normed-fit index (NFI) >0.90, root-mean-square residual (RMR) <0.08, and root-mean-square error of the approximation (RMSEA) <0.08 [28-31]. If required, model modification was performed using modification indices (MI), and the *χ*^2^ statistical difference with one degree of freedom [32]. The sample size required for CFA is 10–20 times the number of observed (measured) variables [33]. The sample size of the present study satisfied this criterion. Internal-consistency reliability was considered to be present when Cronbach’s alpha was ≥0.70 [31]. Test–retest reliability was evaluated using the intraclass correlation coefficient (ICC) with a criterion of ≥0.70 [27]. Convergent validity was evaluated using Pearson’s correlation, and known-groups validity was analyzed using the *t*-test and Cohen’s effect size.

## Results

### Data quality of all 20 items

There was very low rate of missing data for items 1, 2, and 6 (0.2%–0.5%). Among all 20 items, the highest score was 2.2 ± 1.3 (item 12: “Worrying about the future and the possibility of serious complications”) and the lowest score was 0.3 ± 0.7 (item 15: “Feeling unsatisfied with your diabetes physician”; Table 1). The mean scores for the K-PAID and K-PAID-5 were 35.05 ± 21.64 and 8.82 ± 5.55, respectively.

**Table 1:** **Data quality of all 20 items of the K-PAID**

**Item no.**	**Abbreviated item content**	**Percentage of missing values**	**Item score (mean ± SD)**
1	Concrete goals	0.5	1.5 ± 1.3
2	Discouraged	0.2	1.0 ± 1.1
3	Scared	0.0	1.8 ± 1.4
4	Social situations	0.0	1.6 ± 1.4
5	Deprivation	0.0	1.9 ± 1.4
6	Depressed	0.2	1.6 ± 1.4
7	Indistinguishable mood	0.0	1.2 ± 1.2
8	Overwhelmed	0.0	1.6 ± 1.3
9	Reactions	0.0	1.3 ± 1.3
10	Angry	0.0	1.4 ± 1.4
11	Concerned	0.0	2.0 ± 1.3
12	Worry about the future	0.0	2.2 ± 1.3
13	Guilty	0.0	1.7 ± 1.3
14	Accepting	0.0	1.0 ± 1.2
15	Unsatisfied	0.0	0.3 ± 0.7
16	Energy	0.0	1.5 ± 1.3
17	Alone	0.0	0.8 ± 1.2
18	Supportive	0.0	0.9 ± 1.2
19	Coping	0.0	1.7 ± 1.4
20	Burned out	0.0	1.1 ± 1.2

### Factorial-construct validity of the K-PAID and K-PAID-5

As indicated by the data presented in Table 2, the PAID and PAID-5 were previously variously reported to be a one-factor structure [8,16,21], two-factor structure [13,15], three-factor structure [12], and four-factor structure [9]. Based on these empirical findings, CFA was performed in the present study to examine the fit to these predefined models of the K-PAID and K-PAID-5 (Table 3).

**Table 2:** **Underlying structures of the PAID and PAID-5 based on previous psychometric studies including patients with type 2 diabetes**

**Form/authors**	**Structure**	**Scale/subscales and their names (item numbers)**
Full form (PAID)		
Welch et al. [8]	One factor	One-factor scale (all 20 items)
Huang et al. [16]	One factor	One-factor scale (all 20 items)
Miller et al. [13]	Two factors	Subscale 1: Lack of confidence (1, 2, 14, 15, 16, 17, 18)
		Subscale 2: Negative emotional consequences (3, 4, 5, 6, 7, 8, 9, 10, 11, 12, 13, 19, 20)
Huis In ’t Veld et al. [15]	Two factors	Subscale 1: Diabetes distress (1, 2, 3, 4, 5, 6, 7, 8, 9, 10, 11, 12, 13, 14, 19)
		Subscale 2: Support-related issues (15, 16, 17, 18, 20)
Papathanasiou et al. [12]	Three factors	Subscale 1: Diabetes-related emotional problems (1, 2, 3, 6, 7, 8, 9, 10, 12, 13, 14, 15, 16, 19, 20)
		Subscale 2: Food-related problems (4, 5, 11)
		Subscale 3: Social-support-related problems (17, 18)
Snoek et al. [9]	Four factors	Subscale 1: Diabetes-related emotional problems ( 3, 6, 7, 8, 9, 10, 12, 13, 14, 16, 19, 20)
		Subscale 2: Treatment-related problems (1, 2, 15)
		Subscale 3: Food-related problems (4, 5, 11)
		Subscale 4: Social-support-related problems (17, 18)
Graue et al. [19]	Lack of empirical support	
Short form (PAID-5)		
McGuire et al. [21]	One factor	One-factor scale (3, 6, 12, 16, 19)

**Table 3:** **CFA: Goodness-of-fit statistics for the K-PAID and K-PAID-5 models and standardized loadings**

**Underlying factor model**	***χ*** ^**2**^ **(** ***p*** **)**	***df***	**CMIN/DF**	**GFI**	**RMR**	**RMSEA**	**CFI**	**NFI**	**Standardized loadings**
Full form (K-PAID)									
One-factor model^a^	634.567 (*p* < 0.001)	169	3.755	0.864	0.074	0.079	0.903	0.873	0.29–0.83
Two-factor model^b^	615.042 (*p* < 0.001)	168	3.661	0.907	0.074	0.078	0.907	0.877	0.31–0.84
Two-factor model^c^	580.633 (*p* < 0.001)	168	3.456	0.876	0.072	0.075	0.914	0.884	0.31–0.84
Three-factor model^d^	567.558 (*p* < 0.001)	166	3.419	0.878	0.072	0.074	0.917	0.887	0.29–0.83
Four-factor model^e^	561.753 (*p* < 0.001)	164	3.425	0.880	0.072	0.074	0.918	0.888	0.37–0.88
Short form (K-PAID-5)									
One-factor model^f^	11.536 (*p =* 0.021)	4	2.884	0.990	0.039	0.066	0.993	0.989	0.65–0.84

*df,* Degrees of freedom; *CMIN/DF*, Ratio of chi-square value to the degrees of freedom; *GFI*, Goodness-of-fit index; *RMR*, Root-mean-square residual; *RMSEA*, Root-mean-square error of approximation; *CFI*, Comparative-fit index; *NFI*, Normed fit index.

^a^After modification with the covariance of error terms between items 1 and 2 [modification index (MI) = 52.59, ∆*χ* ^2^(1) = 55.989, *p <* 0.01], based on the factor model of Welch et al. [8].

^b^After modification with the covariance of error terms between items 1 and 2 [MI = 51.79, ∆*χ* ^2^(1) = 54.691, *p <* 0.01], based on the factor model of Miller et al. [13].

^c^After modification with the covariance of error terms between items 1 and 2 [MI = 55.56, ∆*χ* ^2^(1) = 55.718, *p <* 0.01], based on the factor model of Huis In ’t Veld et al. [15].

^d^After modification with the covariance of error terms between items 1 and 2 [MI = 53.24, ∆*χ* ^2^(1) = 56.501, *p <* 0.01], based on the factor model of Papathanasiou et al. [12].

^e^Based on the factor model of Snoek et al. [9].

^f^After modification with the covariance of error terms between items 3 and 6 [MI = 31.50, ∆*χ* ^2^(1) = 42.026, *p <* 0.01], based on the factor model by McGuire et al. [21].

For the full-form K-PAID (Table 3), the one-factor model yielded three goodness-of-fit criteria that satisfied the cutoff values (RMR = 0.074, RMSEA = 0.079, and CFI = 0.903) after model modification with the covariance of error terms between items 1 and 2 [∆*χ*^2^(1) = 55.989, *p* < 0.01]. All of the model parameters of this one-factor model were significant at the *p* < 0.05 level, and the standardized factor loadings ranged from 0.29 to 0.83, with the lowest loading value being for item 15 (“Feeling dissatisfied with your diabetes physician”). The two-, three-, and four-factor models yielded very similar findings to those of the one-factor model (Table 3). Overall, the CFAs revealed marginal fits for all of the factor models tested.

For the short-form K-PAID-5, the one-factor model demonstrated excellent goodness-of-fit indices (CMIN/DF = 2.884, GFI = 0.990, RMR = 0.039, RMSEA = 0.066, CFI = 0.993, and NFI = 0.989) after model modification with the covariance of error terms between items 3 and 6 [∆*χ*^2^(1) = 42.026, *p* < 0.01]. The standardized loadings in the one-factor model of the K-PAID-5 ranged from 0.65 to 0.84 (Table 3).

### Internal-consistency reliability of the K-PAID and K-PAID-5

Table 4 indicates that the Cronbach’s alpha of the K-PAID as a one-factor scale was 0.94, which implies excellent internal-consistency reliability. Cronbach’s alpha was calculated for each subscale of the K-PAID, which correspond to the subscales suggested in previous studies. When the K-PAID was structured as two subscales based on previous studies [12,13], the Cronbach’s alpha values were greater than the criterion value of 0.70. However, when the K-PAID was structured as three subscales, the Cronbach’s alpha of the third subscale (“Social-support-related problems”) was 0.61, reflecting unsatisfactory internal-consistency reliability. When the K-PAID was structured as four subscales, the Cronbach’s alpha values of the second (“Treatment-related problems”) and fourth (“Social support-related problems”) subscales were unsatisfactory, at 0.54 and 0.61, respectively.

**Table 4:** **Internal-consistency reliability, test–retest reliability, and convergent validity of the K-PAID and K-PAID-5**

**Underlying structure**	**Scale/subscales and their names (number of items)**	**Cronbach’s alpha**	**ICC (95% confidence interval)**	**Pearson’s** ***r***	
				**CES-D**	**HbA1c**
Full form (K-PAID)					
One-factor structure^a^	Single scale (20 items)	0.94	0.89 (0.83–0.94)	0.55*	0.13*
Two-factor structure^b^	Subscale 1: Lack of confidence (7 items)	0.74	0.85 (0.75–0.91)	0.52*	0.15*
	Subscale 2: Negative emotional consequences (13 items)	0.94	0.90 (0.83–0.94)	0.53*	0.12*
Two-factor structure^c^	Subscale 1: Diabetes distress (15 items)	0.93	0.90 (0.84–0.94)	0.52*	0.13*
	Subscale 2: Support-related issues (5 items)	0.77	0.87 (0.76–0.92)	0.57*	0.13*
Three-factor structure^d^	Subscale 1: Diabetes-related emotional problems (15 items)	0.93	0.89 (0.82–0.93)	0.55*	0.13*
	Subscale 2: Food-related problems (3 items)	0.73	0.80 (0.66–0.88)	0.41*	0.13*
	Subscale 3: Social-support-related problems (2 items)	0.61	0.81 (0.68–0.89)	0.48*	ns
Four-factor structure^e^	Subscale 1: Diabetes-related emotional problems (12 items)	0.94	0.88 (0.81–0.93)	0.54*	0.12*
	Subscale 2: Treatment-related problems (3 items)	0.54	0.30 (–0.18–0.59)	0.32*	0.14*
	Subscale 3: Food-related problems (3 items)	0.74	0.79 (0.66–0.88)	0.41*	0.13*
	Subscale 4: Social-support-related problems (2 items)	0.61	0.81 (0.67–0.89)	0.48*	ns
Short form (K-PAID-5)					
One-factor structure^f^	Single scale (5 items)	0.87	0.89 (0.82–0.94)	0.48*	0.14*

^a^Based on the factor construct of Welch et al. [8]; ^b^Based on the factor construct of Miller et al. [13]; ^c^Based on the factor construct of Huis In ’t Veld et al. [15]; ^d^Based on the factor construct of Papathanasiou et al. [12]; ^e^Based on the factor construct of Snoek et al. [9]; ^f^Based on the factor construct of McGuire et al. [21]; *CES-D*, Center for epidemiologic studies-depression; *HbA1c*, Hemoglobin A1c; *p < 0.01; *ns*, Not statistically significant

The Cronbach’s alpha value of the K-PAID-5 as a one-factor scale was 0.87, demonstrating satisfactory internal-consistency reliability.

### Test–retest reliability of the K-PAID and K-PAID-5

The ICC of the K-PAID as a one-factor scale was 0.89 [95% confidence interval (CI) = 0.83–0.94], indicating the presence of satisfactory temporal stability (Table 4). The ICC of each subscale of the K-PAID was also calculated based on the predefined subscales in previous studies. The ICCs of all of the subscales except for “Treatment-related problems” met the criterion value of 0.70, as also reported by Snoek et al. [9].

The ICC of the K-PAID-5 was 0.89 (95% CI = 0.82–0.94), implying good temporal stability.

### Convergent validity of the K-PAID and K-PAID-5

The overall score of the CES-D was 16.18 ± 9.53 (range = 0–51). As hypothesized, the K-PAID as a one-factor scale was moderately correlated with the CES-D (*r* = 0.55, *p* < 0.001; Table 4). In most cases the Pearson’s correlation coefficients of each subscale relative to the CES-D indicated the presence of moderate correlations. However, the subscale of “Treatment-related problems” was weakly correlated (*r* = 0.32, *p* < 0.001). The K-PAID-5 was moderately correlated with the CES-D score (*r* = 0.48, *p* < 0.001), reflecting satisfactory convergent validity.

The HbA1c level was 7.7 ± 1.4% (60 ± 15 mmol/mol). The K-PAID as a one-factor scale was positively (but weakly) correlated with the HbA1c level (*r* = 0.13, *p* < 0.001), as hypothesized. All subscales of the K-PAID listed in Table 4 were also significantly correlated with the HbA1c level, with the sole exception of “Social-support-related problems” in the three- and four-factor structures. The K-PAID-5 was positively (but again weakly) correlated with the HbA1c level (*r* = 0.14, *p* < 0.001).

### Known-groups validity of the K-PAID and K-PAID-5

As presented in Table 5, the score of the K-PAID as a one-factor scale did not differ significant between males (33.21 ± 22.23) and females (36.99 ± 21.96; *t* = –1.84, *p* = 0.07). When the total K-PAID was separated into two, three, and four subscales, only the mean scores of the “Negative emotional consequences,” “Support-related issues,” and “Diabetes-related emotional problems” subscales differed significantly according to gender, and their Cohen’s effect sizes (*d*) were small, ranging from 0.22 to 0.23. Overall, the known-groups validity of the K-PAID by gender was fair.

**Table 5:** **Known-groups validity of the K-PAID and K-PAID-5 by gender**

**Underlying construct**	**Scale/subscales and their names (number of items)**	**Males (** ***n*** **= 226)**	**Females (** ***n*** **= 214)**	***t*** **(** ***p*** **)**	**Cohen’s effect size (** ***d*** **)**
		**Mean ± SD**	**Mean ± SD**		
Full form (K-PAID)					
One-factor construct^a^	Single scale (20 items)	33.21 ± 22.23	36.99 ± 21.96	–1.84 (*p* = 0.07)	
Two-factor construct^b^	Subscale 1: Lack of confidence (7 items)	8.65 ± 6.21	8.88 ± 6.44	–0.38 (*p* = 0.71)	
	Subscale 2: Negative emotional consequences (13 items)	24.56 ± 16.01	28.11 ± 16.57	–2.29 (*p* = 0.02)	0.22
Two-factor construct^c^	Subscale 1: Diabetes distress (15 items)	28.05 ± 17.40	30.70 ± 17.42	–1.60 (*p* = 0.11)	
	Subscale 2: Support-related issues (5 items)	5.16 ± 4.74	6.29 ± 5.43	–2.32 (*p* = 0.02)^g^	0.22
Three-factor construct^d^	Subscale 1: Diabetes-related emotional problems (15 items)	24.65 ± 16.29	27.64 ± 16.84	–1.89 (*p* = 0.06)	
	Subscale 2: Food-related problems (3 items)	6.60 ± 4.23	6.94 ± 4.16	–0.84 (*p* = 0.40)	
	Subscale 3: Social-support-related problems (2 items)	1.95 ± 2.28	2.41 ± 2.69	–1.93 (*p* = 0.05)^g^	
Four-factor construct^e^	Subscale 1: Diabetes-related emotional problems (12 items)	20.87 ± 14.56	24.40 ± 15.57	–2.46 (*p* = 0.01)	0.23
	Subscale 2: Treatment-related problems (3 items)	3.78 ± 2.94	3.24 ± 2.78	2.00 (*p* = 0.05)	
	Subscale 3: Food-related problems (3 items)	6.60 ± 4.23	6.94 ± 4.16	–0.84 (*p* = 0.40)	
	Subscale 4: Social-support-related problems (2 items)	1.95 ± 2.29	2.41 ± 2.69	–1.93 (*p* = 0.05)^g^	
Short form (K-PAID-5)					
One-factor construct^f^	Single scale (5 items)	7.97 ± 5.35	9.71 ± 5.64	–3.30 (*p* < 0.01)	0.31

^a^Based on the factor construct of Welch et al. [8]; ^b^Based on the factor construct of Miller et al. [13]; ^c^Based on the factor construct of Huis In’t Veld et al. [15]; ^d^Based on the factor construct of Papathanasiou et al. [12]; ^e^Based on the factor construct of Snoek et al. [9]; ^f^Based on the factor construct of McGuire et al. [21]; ^g^Equal variances not assumed.

The mean scores of the K-PAID-5 were significantly higher for females than for males (*t* = –3.30, *p* = 0.001, Cohen’s *d* = 0.31), reflecting satisfactory known-groups validity.

## Discussion

In this study, the full and short forms of the PAID were translated into Korean, and the psychometric properties of these translated scales were evaluated in Korean patients with type 2 diabetes. This is the first study to simultaneously evaluate the psychometric properties of the full and short forms of the instrument in the same sample.

As a one-factor structure, the K-PAID-5 exhibited excellent reliability and validity. However, the factorial structure of the K-PAID identified using CFA revealed a marginal fit, regardless of whether the underlying structure being tested was a one-, two-, three-, or four-factor model. The CFA for the K-PAID also revealed covariance of error terms between item 1 (“Do not have clear and concrete goals for my diabetes care”) and item 2 (“Feel discouraged with my diabetes treatment plan”) on all of the factor models of the K-PAID except the four-factor model reported by Snoek et al. [9]. Similarly, Graue et al. [19] noted in a study of Norwegian patients with diabetes that there was error covariance between items 1 and 2. In general, error covariance may occur when items are difficult to understand [34], and some of the patients interviewed in the present study did experience difficulty comprehending both items 1 and 2. Polonsky et al. [35] also noted that the exact meaning of item 1 was unclear. Considering these findings, items 1 and 2 should be rewritten to ensure that their meanings are unequivocally clear to patients with diabetes.

In this study, the subscale of “Treatment-related problems” (clustered with items 1, 2, and 15) of the K-PAID yielded the lowest Cronbach’s alpha of 0.52, which implies relative heterogeneity among these three items [36]. An unsatisfactory Cronbach’s alpha (0.60) was also found for this subscale in a study involving Brazilian patients [37] that used the four-factor structure of the PAID. This low Cronbach’s alpha, which was anticipated because of the aforementioned comprehension problems with items 1 and 2, may have led to a measurement error that reduced the Cronbach’s alpha of the subscale. Item 15 (“Feeling unsatisfied with your diabetes physician”) yielded a very low score (0.30 ± 0.73) in the present study, implying that too many patients strongly responded to this item with “Not a problem.” This distribution of the scores for item 15 may have been responsible for the finding of poor reliability [27]. Similar distributions of scoring for item 15 were found in patients from Greece (0.36 ± 0.61) [12] and Taipei (0.04 ± 0.27) [16]. The subscale of “Treatment-related problems” was also unsatisfactory with respect to test–retest reliability in the present study. This temporal instability of the subscale is consistent with the finding of Snoek et al. [9].

The subscale of “Social-support-related problems” comprised only two items: item 17 (“Feeling alone with your diabetes”) and item 18 (“Feeling that your friends and family are not supportive of your diabetes management efforts”). The Cronbach’s alpha of this subscale was at the cutoff value (0.70) in a study by Papathanasiou et al. [12], and around the cutoff value (0.69–0.72) in the study by Snoek et al. [9]; in the present study, the Cronbach’s alpha of this subscale was a little lower, at 0.61. When considering that at least three items are needed for a latent subscale [38], either additional items are needed for the subscale of “Social-support-related problems,” or else the entire subscale should be deleted.

Regarding convergent validity, previous studies have demonstrated moderate correlations between the PAID and CES-D (*r* = 0.58–0.45) [13,15], which is consistent with the findings of the present study, with the exception of the second subscale (“Treatment-related problems”) in the four-factor structure of the K-PAID. This exception may be due to the aforementioned lack of reliability of the subscale. As hypothesized, the K-PAID versions were positively but weakly associated with the HbA1c level in the present study, with the exception of the “Social-support-related problems” subscale, for which the Cronbach’s alpha was inadequate. This finding is consistent with previous studies finding greater diabetes-related emotional distress to be correlated with poor glycemic control (*r* = 0.15–0.34) [12,16,24]. The weak magnitude of the association may be attributable to the indirect effect of the diabetes-related emotional distress on the serum HbA1c level through diabetes self-care cactivities [39].

The PAID was originally developed only for female patients with diabetes.^3^ It was subsequently applied to both male and female patients with diabetes, and the effect of gender on the PAID scores was also investigated, revealing differences [15,19] with small-to-moderate effect sizes (Cohen’s *d* = 0.39) [37]. Assessment of the known-groups validity in the present study revealed a gender difference for the K-PAID-5, reflecting satisfactory known-groups validity. However, since the effect size was only small to moderate (Cohen’s *d* = 0.31), this finding must be interpreted with caution.

This psychometric study was subject to a few limitations. One limitation was that it did not examine responsiveness, which is the ability to detect change [27]. Therefore, a longitudinal study with repeated measures is required in which changes in the K-PAID or K-PAID-5 are anticipated to occur. Alterations in the patient’s PAID score are considered to be one of the good outcomes of diabetes education [40]. It is thus recommended that the ability to detect changes in patients using the K-PAID and K-PAID-5 should be assessed before and after administering an education program. For example, Keers et al. [41] offered a diabetes education program to patients, and measured the PAID as an outcome before the program was offered and at 1 year after the program was completed. The effect size of the total PAID was large; that of the subscale of “Treatment-related problems” was small to moderate when the PAID was clustered into four subscales, as reported by Snoek et al. [9]. Another limitation of the present study was the use of a convenience sampling method to recruit participants, which might restrict the ability to generalize the findings to patients with type 2 diabetes.

For further investigations, a systematic review of measurement properties (reliability, validity, and responsiveness) of all studies of the PAID and PAID-5 is recommended. Previous systematic reviews have mainly included randomized clinical trials, observational studies, and diagnostic studies. Systematic reviews focusing on the measurement properties of one or more instruments have rarely been conducted due to the lack of a standard method for assessing the methodological quality of studies that have investigated measurement properties [27]. However, a standard assessment has recently been developed (COSMIN: COnsensus-based Standards for the selection of health Measurement INstruments) [42]. Using this standard assessment, a systematic review of the studies of the PAID and PAID-5 may produce evidence-based information about the appropriateness or the low quality of each of the measurement properties of the PAID and PAID-5, and help guide the agenda for further research on the measurement properties of these instruments.

## Conclusions

The overall psychometric properties of the one- and two-factor structures of the K-PAID were marginally acceptable, and those of the three- and four-factor structures were partially satisfied. Therefore, clinicians and researchers who use the K-PAID need to carefully consider whether they calculate the K-PAID score as a total or as subscales. Meanwhile, the K-PAID-5 demonstrated excellent psychometric properties as a one-factor scale, with satisfactory factorial-construct, convergent, and known-groups validity, and internal-consistency and test–retest reliability. The briefness of the K-PAID-5 may impose a lower burden on patients with type 2 diabetes than the full form of the instrument.

## Abbreviations

PAID: Problem areas in diabetes scale
PRO: Patients-reported outcome
EFA: Exploratory factor analysis
CFA: Confirmatory factor analysis
PAID-5: Problem areas in diabetes scale – Five-item short form
OHA: Oral hypoglycemic agent
K-PAID-5: Korean version of the PAID-5
K-PAID: Korean version of the PAID
CES-D: Center for epidemiologic studies-depression
HbA1c: Hemoglobin A1c
CMIN/DF: Ratio of chi-square value to the degree of freedom
GFI: Goodness fit index
CFI: Comparative fit index
NFI: Normed fit index
RMR: Root-mean-square residual
RMSEA: Root-mean-square error of approximation
MI: Modification indices
ICC: Intracless correlation coefficient
CI: Confidence interval
COSMIN: COnsensus-based Standards for the selection of health Measurement INstruments
